# Supplementary material for: The interactome of intact mitochondria by cross-linking mass spectrometry provides evidence for coexisting respiratory supercomplexes
Source: Mol Cell Proteomics. 2017 Dec 8;17(2):216–32. doi: 10.1074/mcp.RA117.000470 (PMC5795388; doi:10.1074/mcp.RA117.000470)
Supplement: Supplemental Data [file supp_RA117.000470_133922_0_supp_23978_fzffwf.zip › spectra_annotation/mito_DR_spectra_annotation/361-1-4-1-4-1.pdf]

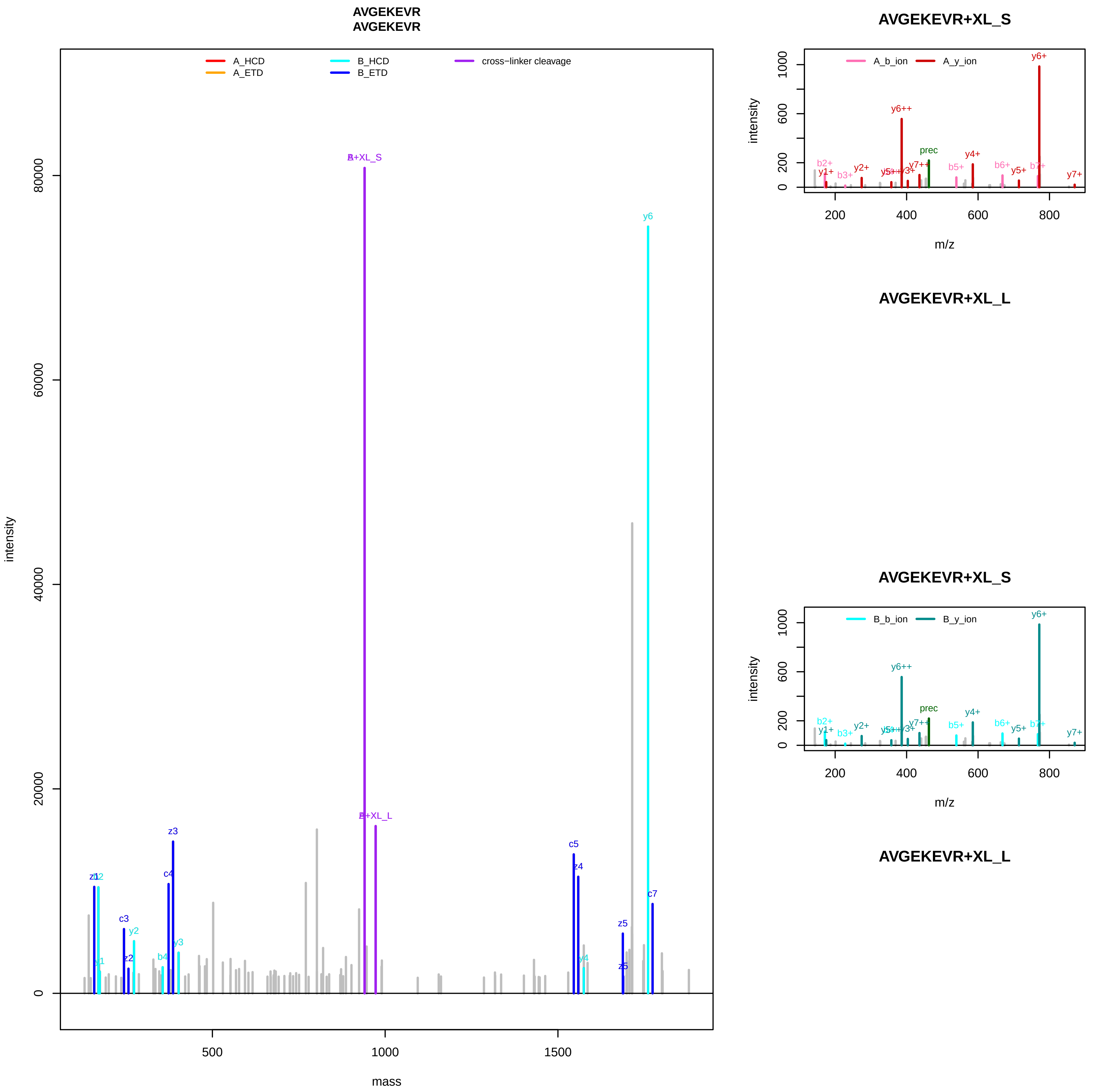
AVGEKEVR
AVGEKEVR
A_HCD
A_ETD
B_HCD
B_ETD
cross−linker cleavage
intensity
mass
0
20000
40000
60000
80000
500
1000
1500
A+XL_S
A+XL_L
y6
z1
y2
y1
c3
z2
y2
b4
y3
c4
z3
c5
z4
y4
z5
z5
c7
AVGEKEVR+XL_S
A_b_ion
A_y_ion
intensity
m/z
0
200
600
1000
200
400
600
800
y6+
y6++
prec
y4+
b2+
y1+
b3+
y2+
y5++
y3+
y7++
b5+
b6+
y5+
b7+
y7+
AVGEKEVR+XL_L
AVGEKEVR+XL_S
B_b_ion
B_y_ion
intensity
m/z
0
200
600
1000
200
400
600
800
y6+
y6++
prec
y4+
b2+
y1+
b3+
y2+
y5++
y3+
y7++
b5+
b6+
y5+
b7+
y7+
AVGEKEVR+XL_L
